# Assessment of Immune Response and Efficacy of Essential Oils Application on Controlling Necrotic Enteritis Induced by *Clostridium perfringens* in Broiler Chickens

**DOI:** 10.3390/molecules26154527

**Published:** 2021-07-27

**Authors:** Mohammad H. Gharaibeh, Mohammad S. Khalifeh, Adi N. Nawasreh, Wael M. Hananeh, Mofleh S. Awawdeh

**Affiliations:** 1 Department of Basic Veterinary Medical Science, Faculty of Veterinary Medicine, Jordan University of Science and Technology, P.O. Box 3030, Irbid 22110, Jordan; mskn@just.edu.jo (M.S.K.); adi.nawasreh@yahoo.com (A.N.N.); 2 Department of Pathology and Public Health, Faculty of Veterinary Medicine, Jordan University of Science and Technology, P.O. Box 3030, Irbid 22110, Jordan; whananeh@just.edu.jo (W.M.H.); mawawdeh@just.edu.jo (M.S.A.)

**Keywords:** necrotic enteritis, *Clostridium perfringens*, essential oils, cytokines, anti-inflammatory pathway, broiler chickens

## Abstract

Necrotic enteritis (NE) caused by *Clostridium perfringens* is one of the most important enteric diseases in poultry. The antibacterial activity of two different essential oil (EO) blends against *C. perfringens* was investigated both in vitro and in vivo. Additionally, the immunological response to EO treatment was assessed. In the in vitro study, the antibacterial activity of EO formulas and commonly used antibiotics was evaluated against *C. perfringens* using disk diffusion assay, minimum inhibitory concentration (MIC) assay, and minimum bactericidal concentration (MBC) assay. In the in vivo study, NE experimental infection was performed on 440 Ross broiler chicks at 19 days of age for 4 continuous days. The chicks were treated with either EOs or amoxicillin at 22 days of age for 5 continuous days. One day after the end of treatment, the birds’ performance was evaluated by calculating the feed conversion ratio. Serum samples from 120 birds were collected to measure the levels of IL-1β, IFN-γ, IL-8, IL-10, and IL-17. After that, all birds were slaughtered, and their small intestines were subjected to gross and histopathological evaluation. In addition, bacterial counts in the small intestines were evaluated. In the in vitro study, EOs showed higher antimicrobial activities in comparison with antibiotics against *C. perfringens*. In the in vivo study, birds treated with EOs showed a significant decrease in bacterial counts, a significant decrease in intestinal lesions, and a significant improvement in performance compared with untreated birds (*p* < 0.05). Moreover, treating birds with EOs directed the immune system toward an anti-inflammatory pathway. None of the treated birds died due to NE compared with the 10% mortality rate in untreated birds. In conclusion, EOs might be an effective and safe alternative to antibiotics in the treatment of chicken NE.

## 1. Introduction

Necrotic enteritis (NE) is one of the most important enteric diseases in poultry as it causes huge economic losses in the poultry industry, which could come close to USD 6 billion per year worldwide for medical treatments and impaired chicken performance [1]. The main causative agent of NE disease is *C. perfringens*, which produces a novel toxin (NetB), which is a key virulence factor for experimental NE disease development [2]. *C. perfringens* is an opportunistic Gram-positive anaerobic bacterium that is normally present in the intestines of healthy humans and animals [3], but under certain abnormal conditions, *C. perfringens* may overgrow and multiply, which leads to NE development [4]. *C. perfringens* carries public threats to human health due to possible contamination of chicken products prepared for human consumption. *C. perfringens* is the second most common cause of foodborne disease in the US by causing about 1 million illnesses annually [5].

Traditionally, NE has been controlled by antimicrobial growth promoters (AGPs) [6]. The ban of AGP use in many European countries has had a bad effect on the colonization of *C. perfringens* and the prevention of necrotic enteritis in poultry, and the misuse of antibiotics has led to an increase in the prevalence and risk of antibiotic resistance of *C. perfringens* infection in broiler chickens [7]. Therefore, there has been a growing interest in alternative antimicrobial agents for controlling NE disease. Essential oils (EOs) have been used in treating many diseases because they have potent antimicrobial activities [8,9,10,11,12,13] and anti-inflammatory effects [14,15,16]. Recently, several studies have shown the positive impact of EOs on NE caused by *C. perfringens* in chickens [17,18]. Therefore, there has been an increasing interest in finding alternatives for growth promoters and strengthening of the immune system [19]. The aim of this study was to investigate the antibacterial and immunological activities of two EO blends (provided by Animal Wellness Products, Modena, Italy) against a NetB-carrying isolate of *C. perfringens* both in vitro and in vivo in comparison with commonly used antibiotics.

## 2. Results

### 2.1. Disk Diffusion Assay

The results of the disk diffusion assay are shown in Table 1. Both EOs D and E gave the largest inhibition zone among all treatments. Amoxicillin gave the largest inhibition zone among the antibiotics but not a larger inhibition zone than those of EOs D and E.

### 2.2. Minimal Inhibitory Concentration Assay (MIC) and Minimum Bactericidal Concentration Assay (MBC)

The obtained results for MICs and MBCs are shown in Table 2. *C. perfringens* showed higher susceptibility to EOs D and E than other EOs.

### 2.3. Chicks’ Performance

**Figure 1:**
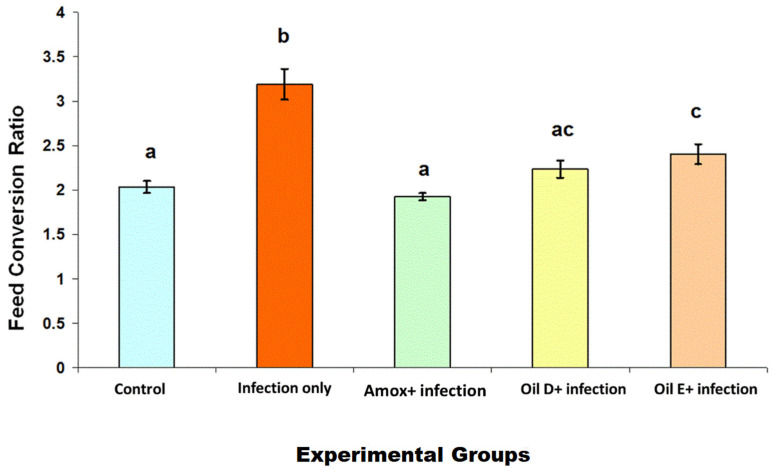
Feed conversion ratio in birds in response to NE caused by *C. perfringens* infection. The feed conversion ratio was calculated by dividing the average feed intake over the average weight gain per replicate per group. The values are means ± S.E. Different letters represent statistically significant differences with a *p*-value less than 0.05.

The results of feed conversion ratio are shown in Figure 1. Groups that were treated with either EO D, EO E, or amoxicillin showed a significant decrease in feed conversion ratio (*p* < 0.05) compared with the infected untreated group. There was no significant difference between the EO groups (*p* < 0.05), while a significant difference was observed between the EO E group and the amoxicillin group (*p* < 0.05) and between the EO E group and the control groups (noninfected and untreated groups).

### 2.4. Bacterial Count

**Figure 2:**
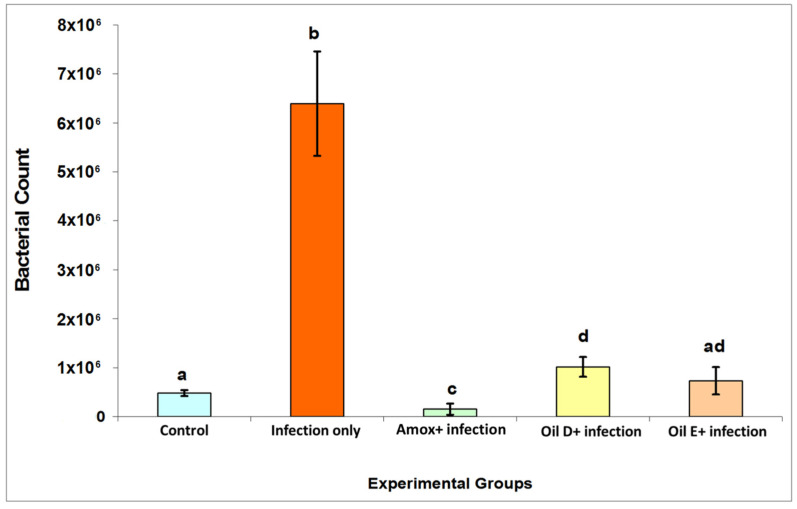
*C. perfringens* count was recovered from the intestinal contents of the birds with and without NE. All the control groups reflect the basal bacterial counts in the presence of intestinal stressors (nutritional and coccidial overdose vaccination) in the untreated nonexperimentally infected birds. The values are means ± S.E. Different letters represent statistically significant differences with a *p*-value less than 0.05.

Bacterial count results are presented in Figure 2. The infected untreated group showed an increase in bacterial count compared with all the experimental groups (*p* < 0.05). There was no significant difference between the two groups that received EOs (*p* < 0.05). Amoxicillin decreased the bacterial count significantly when compared with all the other groups (*p* < 0.05). It was also observed that there was no significant difference between the EO E and control groups (*p* < 0.05).

### 2.5. Immunological Response

**Figure 3:**
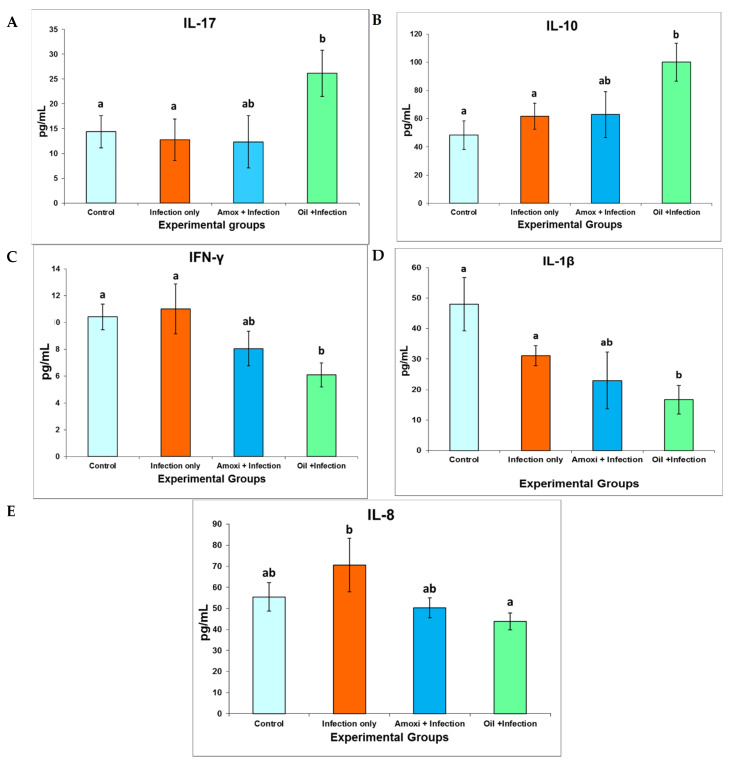
The concentrations of the IL-17 (**A**), IL-10 (**B**), IFN-γ (**C**), IL-1β (**D**), and IL-8 (**E**) cytokines in comparison with the challenge experimental NE study obtained through ELISA. At 19 days of age, the chickens were infected for 4 continuous days through oral administration of broth cultures of *C. perfringens*. On the second day of *C. perfringens* infection, the chicks were orally administrated the recommended dose of the coccidial vaccine 10 times. The treatment was administrated in drinking water on day 22 and continued for 5 days. Sera were collected at day 27 postchallenge. The values are means ± S.E. Different letters represent statistically significant differences with a *p*-value less than 0.05.

Immunological response was determined by measuring the IL-1β, IFN-γ, IL-8, IL-10, and IL-17 levels in chicks’ serum using ELISA kits. We dealt with the two EOs as one unit because we found that there was no significant difference between them in effecting the immune response (*p* < 0.05). IL-17 level is shown in Figure 3A. The birds treated with EOs showed a significant increase in IL-17 level in comparison with the control and infected untreated groups (*p* < 0.05). IL-10 level is shown in Figure 3B. The birds treated with EOs showed a significant increase in IL-10 level in comparison with the control and infected untreated groups (*p* < 0.05). IFN-γ level is shown in Figure 3C. The birds treated with EOs experienced a significant decrease in IFN-γ level in comparison with the control and infected untreated groups (*p* < 0.05). IL-1β level is shown in Figure 3D. The birds treated with EOs showed a significant decrease in IL-1β level in comparison with the control and infected untreated groups (*p* < 0.05). IL-8 level is shown in Figure 3E. The birds treated with EOs experienced a significant decrease in IL-8 level in comparison with the infected untreated group (*p* < 0.05).

### 2.6. Gross Lesions

**Figure 4:**
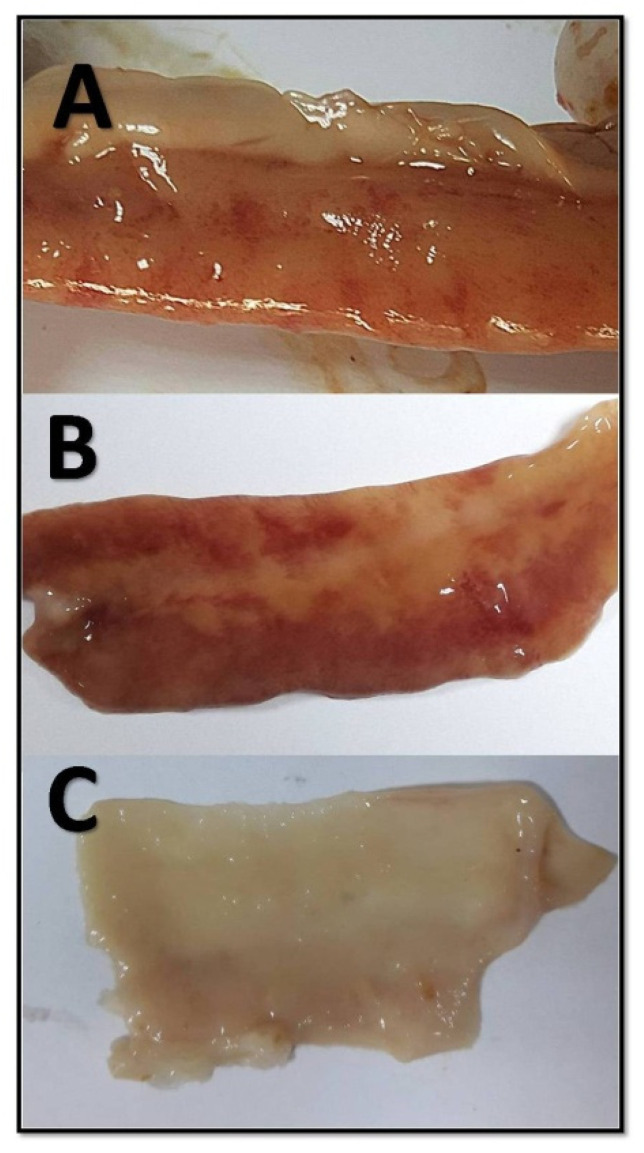
Different lesions of NE in the small intestine. (**A**) Thickened mucus, variable degrees of serosal blood vessel congestion. (**B**) Hemorrhage of the intestinal wall, fibrin deposition, and necrotic debris of the mucus membrane. (**C**) No gross lesions are present.

**Figure 5:**
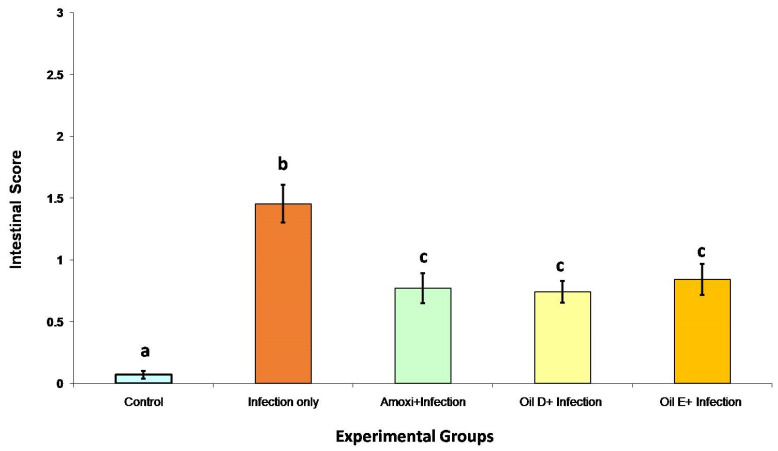
Overall NE lesion score for the entire length of the small intestine. The score sum for all the separate intestinal portions (duodenum, jejunum, and ileum) ranges from 0 to 3. All the control groups reflect the basal intestinal scores in the presence of intestinal stressors (nutritional and coccidial overdose vaccination). The values are means ± S.E. Different letters represent statistically significant differences with a *p*-value less than 0.05.

The main lesion features that were adopted by this study are the presence of ulcer in the intestinal mucosa with acute bright-red hemorrhage within the ulcer bed with or without crusting in all parts of the small intestine with brown fluid. The entire length of the small intestine was checked for any lesion, and each part of the small intestine (duodenum, jejunum, and ileum) was evaluated according to Figure 4. Overall, the NE lesion score was measured by calculating the sum for all the separate intestinal portions (duodenum, jejunum, and ileum) (normal = 0, abnormal = 1); the scores ranged from 0 to 3. The overall NE lesion score for the entire length of the small intestine is shown in Figure 5 The groups treated with either EO D, EO E, or amoxicillin showed a significant decrease in intestinal lesion score compared with the infected untreated group with no significant difference between these treatments (*p* < 0.05). The control groups had the lowest intestinal lesion score among all the other groups (*p* < 0.05).

## 3. Discussion

The current work was designed to investigate the antibacterial efficacy of two EO blends in the treatment of *C. perfringens* infection in broilers, focusing on their effects on growth performance, bacterial count, gross lesion, and immunological profile. *C. perfringens* is one of the most deleterious pathogens in the poultry industry worldwide [1]. *C. perfringens* is an opportunistic bacterium that is present normally in the intestines of healthy broiler chickens with low count (up to 10^4^ CFU/g), but under certain conditions, *C. perfringens* may multiply and overgrow, leading to an increase in bacterial count (up to 10^7^ to 10^8^ CFU/g), and cause clinical disease [20]. In the current study, we found a significant increase in *C. perfringens* count in the intestines of the birds in the infected untreated group compared with those in the control groups (*p* < 0.05). As a result of EO treatment, treating the birds with either EO D or E showed a clear decrease in bacterial count in the intestines of the chickens in comparison with those of the infected untreated birds (*p* < 0.05). In the current study, all the control groups showed a level of *C. perfringens* count in the chickens’ intestines, which seems not to cause any pathological changes. Upon model induction and in the presence of intestinal stressors, an increase in bacterial count was clearly observed. This was possibly due to placing the chickens on a high protein diet [20] and the administration of the recommended dose of coccidial vaccine 10 times [21].

Growth-promoting antibiotics have been used for many decades in broilers’ feed to enhance chicks’ performance. However, the banning of the use of growth-promoting antibiotics in many European Union countries has led to an increase in the prevalence of *C. perfringens* infection in broiler chicks and an increase in outbreaks of NE [22]. As a result, there is a global trend toward searching for alternative treatments, such as EOs [8,9,10,11,16], to limit the emergence and spread of bacterial infections. NE disease provokes lesions and changes in the integrity of the intestine. A study in the US ranged the gross lesions in the small intestine of diseased chickens with NE from superfine and friable to frank and extensive necrotic lesions [23]. In the current study, we scored the gross lesions in the small intestine of diseased chickens with NE from 0 to 3, which are represented in Figure 4. Necrotic lesion severity was negatively correlated with the EO D and E treatments that were introduced to chickens for 5 days after the last dose of *C. perfringens*.

Birds’ performance is represented by the feed conversion ratio. As the costs of broiler feed represent about 75% of the total cost of broiler production [24], the feed conversion efficiency is considered one of the important parameters for broiler production. *C. perfringens* is a major concern in the poultry industry because it causes economic losses by decreasing birds’ performance by increasing the feed conversion ratio and impairing nutrient absorption due to intestinal mucosal damages [25]. The current results support these findings by showing a significant increase in feed conversion ratio in the infected untreated group compared with the control groups (*p* < 0.05). Using EO D, EO E, and amoxicillin as treatments decreased the feed conversion ratio significantly compared with the infected untreated group (*p* < 0.05).

In order to explain the mechanism of EOs’ effect on NE, immune response was evaluated in all the experimental groups by measuring several key inflammatory and regulatory cytokines. An enhancement in the gene expression levels of IL-17, which is produced by Th17 cells as a proinflammatory cytokine following NE infection, has been found [26]. On the contrary, Th17 cells that produce IL-17 with IL-10 do not induce tissue inflammation; rather, they may have an anti-inflammatory effect, leading to alleviating the inflammation [27]. This correlation between IL-17 and IL-10 has not been studied in chickens with NE disease. In the present work, we are the first to study the correlation between IL-17 and IL-10 in chickens with NE disease. We found that treating birds with EOs directs the immune system toward an anti-inflammatory pathway by increasing the levels of both IL-17 and IL-10 significantly, compared with the infected untreated and control groups (*p* < 0.05).

The IFN-γ, IL-1β, and IL-8 cytokines were found to be upregulated in chickens following NE infection [15,28,29] as pro-inflammatory cytokines, which lead to tissue inflammation [30,31,32]. In the current study, treating birds with EOs alleviated the intestinal inflammation through a decrease in the level of the IFN-γ, IL-1β, and IL-8 cytokines compared with the infected untreated and control groups (*p* < 0.05).

NE causes poor performance due to a decrease in feed digestion and absorption as a result of intestinal mucosal damage. Fibrinonecrotic enteritis is the main histopathological finding in chickens with NE [22,33]. In the current study, diseased chickens had severe fibrinonecrotic necrosis with inflammatory cells in all parts of the small intestine. NE disease affects 2- to 5-week-old broiler chickens and can increase the mortality rate by up to 50%, which results in huge economic losses [34]. In the current study, none of the treated birds died due to NE compared with the 10% mortality rate in the infected untreated group. In summary, the current study showed that EO blends significantly reduced bacterial count, lowered the FCR, lowered gross lesions, and decreased intestinal inflammation compared with the controls.

## 4. Materials and Methods

### 4.1. Essential Oil Composition and the Source of Infection Isolate

Six commercial EO blends (A–F) were used to determine their minimum inhibitory concentration (MIC) and MBC. All of the EOs were supplied by Animal Wellness Products, Reggio Emilia, Italy. The chemical compositions of the six EOs were previously presented in Gharaibeh et al. [35]. All oils were stored at 4 °C until used. One *C. perfringens* isolate was obtained from the Department of Pathology and Public Health, Faculty of Veterinary Medicine, Jordan University of Science and Technology. This isolate was selected among *C. perfringens* isolates according to the result of a NetB-carrying toxin. The *C. perfringens* isolate was originally obtained from the intestines of 3- to 5-week-old broiler chicken flocks from Jordan with a history of severe intestinal necrotic lesions [36].

### 4.2. Determination of Disk Diffusion Assay

Disk diffusion assay was performed according to the Clinical and Laboratory Standards Institute (CLSI) [37] with slight modification as previously described by Gharaibeh et al. [35]. Briefly, sterile filter paper discs (Whatman disc, 6 mm diameter) (Oxoid, Basingstoke, UK) were impregnated with 15 µL of the EOs and kept at room temperature for 1 h. Freshly prepared Mueller Hinton broth culture of a *C. perfringens* NetB-positive toxin isolate (used for challenge) was adjusted to 0.5 McFarland turbidity, which is equivalent to 1 × 10^8^ CFU/mL, by using a spectrophotometer (Thermo Fisher Scientific, Waltham, MA, USA) at 600 wavelength with 0.132 reading. Then the cultured broth was streaked evenly into a Mueller Hinton agar medium (Oxoid, UK) using a cotton swab in three directions. The EO discs and the antibiotic discs (Table 1) were applied on the Mueller Hinton agar surface and incubated at 37 °C for 24 h under anaerobic conditions.

### 4.3. Minimal Inhibitory Concentration Assay (MIC) and Minimum Bactericidal Concentration Assay (MBC)

Disk diffusion assay results clearly showed that amoxicillin carried the best anticlostridial activity among the common commercially antibiotics used. Therefore, amoxicillin was selected to be tested using MIC and MBC assays along with two EOs. MIC and MBC against a *C. perfringens* NetB-positive toxin isolate (used for challenge) were achieved in triplicate using broth microdilution according to the CLSI method as previously described by Radaelli et al. [38]. Briefly, fresh *C. perfringens* cultures were grown overnight anaerobically in Reinforced Clostridial broth (Oxoid, UK). Then 50 µL of sterile Reinforced Clostridial broth was added to each well of 96-well microplates. After that, 50 µL of each of the antimicrobial treatments including the six different EO formulas and amoxicillin were added to each well of the first column of 96-well microplates so that each well of the first column contained a different antimicrobial agent. A twofold serial dilution was used to get the final concentration ranging from 256 to 0.000122 µL/mL for each EO formula, and for amoxicillin, from 256 to 0.125 µg/mL. After that, the bacterial broth was adjusted to 0.5 McFarland turbidity, which is equivalent to 1 × 10^8^ CFU/mL, then diluted to 1:100 by adding 0.1 mL of the bacterial broth to 9.9 mL of clean broth to get a concentration of 1 × 10^6^ CFU/mL. Then 50 µL of the suspension broth was added to each well of 96-well microplates with mixing to get a final concentration of 5 × 10^5^ CFU/mL and incubated at 37 °C for 24 h under anaerobic conditions. MIC values were defined as the lowest concentration of the compound that inhibited visible bacterial growth after 24 h of anaerobic incubation. On the other hand, MBC values were determined by culturing 100 µL from the first well showing no growth and two wells before on a tryptose sulfite-cycloserine agar medium (Oxoid, UK) with d-cycloserine supplement (Sigma, Ronkonkoma, NY, USA) anaerobically for 24 h.

### 4.4. Birds and Housing

A total of 440 1-day-old Ross broiler chicks were obtained from a commercial hatchery. The chicks were divided into 10 experimental groups (Table 3). Each group was placed in a separate cage, where it was further divided into four replicates with 11 birds per replicate (1 m^2^). The chicks were raised on antibiotic-free starter feed until 11 days of age. At 11 days old, the feed was changed from starter to grower feed (Table 4). At 19 days old, the chicks were placed on high wheat ration and high fish meal diet (by replacing the soybean meal with fish meal), which was continued for 8 days. This ration is known as a predisposing factor for successful NE model development [20]. The in vivo experiments were carried out according to the recommendations and approval of the Animal Care and Use Committee, Jordan University of Science and Technology (JUST-ACUC).

### 4.5. Challenge Procedure

For the challenge experiment, a pilot study was performed to determine the best bacterial isolate and dose to be used. In the challenge experimental NE study, the infection was induced at 19 days of age for 4 continuous days through oral administration of each chick with 0.5 mL of freshly prepared Mueller Hinton broth cultures of a *C. perfringens* NetB-positive toxin isolate of 1.2 × 10^9^ per mL, while the control chicks were treated similarly but only received broth free of *C. perfringens* as a placebo. On the second day of *C. perfringens* infection, the chicks were orally administrated the recommended dose of coccidial vaccine (Immucox, Ceva, Libourne, France) (Table 4) 10 times to enhance NE disease development [21].

### 4.6. Treatment

Based on the in vitro results, which clearly showed that EO D and E carried the best anticlostridial activity, EO D and E were selected among the six formulas of EOs, and amoxicillin among the common antibiotics commercially used, to be used as treatments in the in vivo study. The concentrations of the EOs used for the in vivo study were determined according to the MBC results. The treatment concentrations were 8 times the MBC value. Therefore, from the in vitro experiments, formula D was used at a dilution factor of 1:2000 from stock (500 µL/L), formula E was tested at a dilution factor of 1:1000 from stock (1000 µL/L), and amoxicillin (Chemifarma S.p.A., Forlὶ, Italy) was used at its highest recommended dose (0.12 g/L). The treatment was administrated in drinking water at 22 days of age (on the day clinical signs first appeared) and continued for 5 days.

### 4.7. Chickens’ Performance

The birds’ performance was evaluated by calculating the feed conversion ratio for all the groups by dividing the average of feed intake (offered feed minus refused feed) per replicate over the average of weight gain for the same replicate and then by calculating the average of all replicates in the same group. The chicks were weighted on the first day of infection and on the day of slaughter (day 27).

### 4.8. Immunological Response

One day after the end of the treatment (day 27), blood samples from 120 birds (3 birds from each replicate) were collected from the wing vein into 1.5 mL Eppendorf tubes, centrifuged at 6000 rpm for 10 min to obtain the serum, and then stored at −20 °C. The serum cytokine (IL-1β, IFN-γ, IL-8, IL-10, and IL-17) titers were measured by commercially available ELISA kits (MyBioSource ELISA kits, San Diego, CA, USA) and according to the manufacture’s protocol.

### 4.9. Bacterial Count

Three birds from each replicate were slaughtered, and bacterial count was performed by weighting 1 g of pooled intestinal contents. The contents (1 g) were then added to 9 mL of peptone water and mixed well, followed by 10-fold serial dilutions (10^−1^–10^−4^). An amount of 100 µL of each diluted tube was cultured on tryptose sulfite-cycloserine agar media (Oxoid, UK) with d-cycloserine (Sigma, USA) and incubated at 37 °C anaerobically for ~48 h. At the end of the incubation period, black bacterial colonies were counted [39].

### 4.10. Gross Lesion Evaluation

All the birds were slaughtered, and their small intestines were subjected to gross lesion evaluation, a procedure that was modified from Cooper and Songer [23]. The presence of one or more of the following changes in the small intestines—thickened mucus, variable degrees of serosal blood vessel congestion (Figure 4A), hemorrhage of the intestinal wall, fibrin deposition, and necrotic debris of the mucus membrane (Figure 4B)—was considered an indicator that a bird was positive for necrotic enteritis. On the other hand, the absence of any significant gross lesions within the small intestines was considered an indicator that a bird was normal (Figure 4C). Birds that died due to the infection were examined and found to have severe necrotic enteritis lesions in all intestinal tissue parts (Figure 4B).

### 4.11. Statistical Analysis

Statistical analysis was performed using OpenEpi (http://www.openepi.com/Menu/OE Menu.htm, accessed on 10 March 2018). The results were compared using a two-way analysis of variance (ANOVA), and significant differences among means were tested using Student’s *t*-test. Only *p*-values less than 0.05 were considered statistically significant.

## 5. Conclusions

EOs decrease NE disease severity by alleviating the inflammation, decreasing the mortality rate, enhancing birds’ performance, decreasing the intestinal *C. perfringens* count, and alleviating intestinal lesions. Therefore, we conclude that EOs are an effective and safe alternative to antibiotics in chickens when treating NE disease.

## Figures and Tables

**Table 1:** Growth inhibition zones of different treatments against *C. perfringens* according to disk diffusion assay.

Treatment	Inhibition Zone (mm)	Source
Essential oil D	52	Animal Wellness Products/Italy
Essential oil E	40	Animal Wellness Products/Italy
Amoxicillin	24	Bioanalyse/Turkey
Doxycycline	18	Arab Co. for Medical Diagnostics/Jordan
Tetracycline	17	Bioanalyse/Turkey
Enrofloxacin	16	Bioanalyse/Turkey
Penicillin	15	Bioanalyse/Turkey
Oxytetracycline	14	Arcomex/Jordan

**Table 2:** MICs and MBCs of different treatments against *C. perfringens*.

Treatment	MIC	MBC
Essential oil A	1.666 ± 0.33 (µL/mL)	NA
Essential oil B	0.833 ± 0.166 (µL/mL)	NA
Essential oil C	1.666 ± 0.33 (µL/mL)	NA
Essential oil D	0.026 ± 0.005 (µL/mL)	0.026 ± 0.005 (µL/mL)
Essential oil E	0.052 ± 0.010 (µL/mL)	0.052 ± 0.010 (µL/mL)
Essential oil F	3.333 ± 0.66 (µL/mL)	NA
Amoxicillin	0.416 ± 0.08 (µg/mL)	0.416 ± 0.083 (µg/mL)

NA: not applicable.

**Table 3:** Experimental groups.

	*C. Perfringens* Challenged	10^X^ Coccidial Vaccine Dose Administration	Treatment/Concentration
Bird age	19–22 days	20 days	22–26 days
Group 1	None	None	None
Group 2	None	Yes	None
Group 3	None	None	Formula D/high
Group 4	None	None	Formula E/high
Group 5	Yes	Yes	None
Group 6	Yes	Yes	Amoxicillin
Group 7	Yes	Yes	Formula D/high
Group 8	Yes	Yes	Formula D/low
Group 9	Yes	Yes	Formula E/high
Group 10	Yes	Yes	Formula E/low

**Table 4:** Feed formula.

Feed Type	Kg per Ton
Wheat	512
Corn	120
Soybean meal	300
Oil	25
Ca Carbonate	9.0
MonoCa Pho	16.3
Met	3.5
Thr	1.0
Lys	2.5
Salt	2.0
Na Bicarb	2.7
Vit/Min	6.0
Total	1000

## Data Availability

All the data generated or analyzed during this study are included in this published article.

## Sample Availability

Samples of the compounds are available from the authors.
